# Proteasome inhibitor bortezomib prevents proliferation and migration of pulmonary arterial smooth muscle cells

**DOI:** 10.1002/kjm2.12835

**Published:** 2024-04-29

**Authors:** Yi‐Ching Liu, Yu‐Hsin Tseng, Yu‐Hsin Kuan, Lin‐Yen Wang, Shang‐En Huang, Siao‐Ping Tsai, Jwu‐Lai Yeh, Jong‐Hau Hsu

**Affiliations:** ^1^ Department of Pediatrics, Kaohsiung Medical University Hospital Kaohsiung Medical University Kaohsiung Taiwan; ^2^ Department of Medical Research, E‐Da Hospital I‐Shou University Kaohsiung Taiwan; ^3^ Department of Pediatrics Chi‐Mei Medical Center Tainan Taiwan; ^4^ School of Medicine, College of Medicine National Sun Yat‐sen University Kaohsiung Taiwan; ^5^ School of Medicine, College of Medicine Kaohsiung Medical University Kaohsiung Taiwan; ^6^ Department of Childhood Education and Nursery Chia Nan University of Pharmacy and Science Tainan Taiwan; ^7^ Department of Pharmacology, College of Medicine Kaohsiung Medical University Kaohsiung Taiwan; ^8^ Department of Pediatrics, School of Medicine, College of Medicine Kaohsiung Medical University Kaohsiung Taiwan; ^9^ Department of Medical Research Kaohsiung Medical University Hospital Kaohsiung Taiwan; ^10^ Graduate Institute of Medicine Kaohsiung Medical University Kaohsiung Taiwan; ^11^ Department of Marine Biotechnology and Resources National Sun Yat‐sen University Kaohsiung Taiwan

**Keywords:** bortezomib, migration, proliferation, pulmonary arterial hypertension

## Abstract

Pulmonary vascular remodeling is a key pathological process of pulmonary arterial hypertension (PAH), characterized by uncontrolled proliferation and migration of pulmonary arterial smooth muscle cells (PASMCs). Bortezomib (BTZ) is the first Food and Drug Administration (FDA)‐approved proteasome inhibitor for multiple myeloma treatment. Recently, there is emerging evidence showing its effect on reversing PAH, although its mechanisms are not well understood. In this study, anti‐proliferative and anti‐migratory effects of BTZ on PASMCs were first examined by different inducers such as fetal bovine serum (FBS), angiotensin II (Ang II) and platelet‐derived growth factor (PDGF)‐BB, while potential mechanisms including cellular reactive oxygen species (ROS) and mitochondrial ROS were then investigated; finally, signal transduction of ERK and Akt was examined. Our results showed that BTZ attenuated FBS‐, Ang II‐ and PDGF‐BB‐induced proliferation and migration, with associated decreased cellular ROS production and mitochondrial ROS production. In addition, the phosphorylation of ERK and Akt induced by Ang II and PDGF‐BB was also inhibited by BTZ treatment. This study indicates that BTZ can prevent proliferation and migration of PASMCs, which are possibly mediated by decreased ROS production and down‐regulation of ERK and Akt. Thus, proteasome inhibition can be a novel pharmacological target in the management of PAH.

## 1 INTRODUCTION

Pulmonary arterial hypertension (PAH), a severe progressive disorder with high morbidity and mortality, is mainly caused by the increase of pulmonary vascular resistance (PVR).^1^, ^2^ The main cause of increased PVR in most patients with PAH is due to fixed vascular obstruction with loss of the cross‐sectional area, which leads to reduced cardiac output, right heart failure, and premature death.^3^ Vasodilators are the mainstay of treatment for PAH; however, vasodilators treatment does not directly improve vascular obstruction nor resolve right ventricular hypertrophy caused by fibrosis, ischemia, and microvascular rarefaction.^4^, ^5^ A new treatment paradigm for PAH is crucially needed to address the severe disease caused by dysfunction of the right ventricle and pulmonary vasculature.

Pulmonary vascular remodeling, a key pathological process of PAH, includes hyperplasia of the media and neomuscularization in the subendothelial layer, driven by uncontrolled proliferation and migration of pulmonary arterial smooth muscle cells (PASMCs).^1^ Oxidative stress, an imbalance between reactive oxygen species (ROS) production and the antioxidant capacity of cells, has also been reported to contribute to the pathogenesis of PAH in several ways, such as pulmonary vascular remodeling, dysfunction of pulmonary endothelial cells, proliferation of PASMCs, and right ventricular hypertrophy. Antioxidant therapy has also become a major area of research in the treatment of PAH.^6^ Several studies have shown that the dysregulation of PASMCs can be triggered by numerous environmental and extracellular factors, such as hypoxia, tumor necrosis factor, angiotensin II (Ang II), platelet‐derived growth factor (PDGF), and endothelial (ET)‐1.^7^ In addition, Ang II and PDGF are considered to be the potent mitogen and chemoattractant for PASMCs and contribute essentially to the progression of PAH.^8^, ^9^

Proteasomes are multimeric protease complexes, comprising a 20S core catalytic complex with 19S regulatory subunits at each end. The 20S core catalytic complex contains three active sites, including chymotrypsin‐like (CT‐L), trypsin‐like (T‐L), and caspase‐like (CP‐L) active sites.^10^ There is emerging evidence showing that proteasome has a role in the pathogenesis of PAH. For example, a proteomic analysis reveals that proteasome subunit beta 6 is involved in pulmonary vascular remodeling in rats and knockdown of this subunit using siRNA prevented PASMC proliferation.^11^ Similarly, a recent animal study of PAH also demonstrated upregulation of proteasome in the mitochondrial proteomic analysis.^12^ The exact mechanisms of proteasome in PAH is not fully understood. However, proteasome inhibition has also been shown to increase anti‐oxidative capacity by increasing antioxidant proteins such as the superoxide dismutase type 1 (SOD1) and catalase, and reducing the nicotinamide adenine dinucleotide phosphate (NADPH) oxidase expression.^13^, ^14^ In addition, proteasome inhibition may also effectively scavenge ROS by increasing the expression and activity of nitric oxide synthase, and promoting nitric oxide production. BTZ is the first Food and Drug Administration (FDA)‐approved proteasome inhibitor for the treatment of multiple myeloma.^15^ It is known that inhibition of proteasome function ameliorates the development of PAH^16^ and proteasome inhibitor inhibits the proliferation and migration of vascular smooth muscle cells.^2^, ^17^, ^18^ BTZ attenuates hypoxia‐induced PASMCs proliferation by restoring mitofusin‐2 expression, and inhibits right ventricular hypertrophy and pulmonary vascular remodeling both in hypoxia‐ and monocrotaline‐induced PAH animal models.^2^, ^19^ Even though anti‐proliferative effects of BTZ in PASMCs has been reported,^19^ the mechanism is not well understood. Furthermore, it remains unclear whether it can inhibit migration, another major factor mediating vascular remodeling.

In this study, the role of BTZ on proliferation, migration, ROS production, and the phosphorylation of ERK and Akt in Ang II‐ and PDGF‐BB‐induced PASMCs models have accordingly been clarified.

## 2 METHODS

### 2.1 Materials and reagents

BTZ (Cat #TRC‐B675700) was obtained from Toronto Research Chemicals (Martin Ross Ave, North York, Canada). Ang II (Cat #HY‐13948) was obtained from MedChemExpress (Monmouth Junction, NJ 08852, USA). PDGF‐BB (Cat #P4056), 3‐[4,5‐dimethylthiazol‐2‐yl]‐2,5‐diphenyl tetrazolium bromide (MTT, Cat #298931), and N‐Acetyl‐L‐cysteine (NAC, Cat #A7250) were obtained from Sigma‐Aldrich, Inc. (St. Louis, MO, USA). 2′,7′‐Dichlorofluorescin diacetate (DCFH‐DA, Cat #C6827) and MitoSOX (Cat #M36008) were purchased from Invitrogen™ (Massachusetts, USA). Antibodies of phosphorylated ERK1/2 (Cat #9101) was obtained from Cell Signaling Technology. (Beverly, MA, USA). Antibodies of ERK1/2 (Cat #06‐182) was obtained from Upstate Biotechnology, Inc. (Lake Placid, NY, USA). Anti‐phospho‐Akt antibody (Cat #GTX128414) and Akt (Cat #GTX121937) were obtained from GeneTex, Inc. (Irvine, CA, USA). Antibody of β‐actin was obtained from Arigo Biolaboratories Corp. (Cat #ARG62346, Hsinchu, Taiwan). Dulbecco's modified Eagle's medium (DMEM), fetal bovine serum (FBS), penicillin, streptomycin, and all other tissue culture reagents were obtained from GIBCO BRL Life Technologies (Grand Island, NY, USA).

### 2.2 Preparation of PASMCs

PASMCs were isolated from the pulmonary artery of 16 male Wistar rats (6‐ to 8‐week‐old; 180–200 g) purchased from BioLASCO Taiwan Co., Ltd. (Taipei, Taiwan). These rats were sacrificed in different experiments. This study was approved by the Institutional Animal Care and Use Committee of the Kaohsiung Medical University (IACUC Approval No: 108118). Animals were cared for in accordance with Guide for the Care and Use of Laboratory Animals published by the United States National Institutes of Health. All rats were housed in a temperature‐controlled environment (22 ± 2°C) with a relative humidity of 55 ± 10% and a 12‐h light/12‐h dark cycle with free access to food and sterile tap water. PASMCs were cultured in DMEM supplemented with 10% FBS, 2 mM glutamine, 100 U/mL penicillin, and 100 mg/mL streptomycin and incubated in a humidified 37°C incubator with 5% CO_2_. Cells were passaged at 70%–80% confluence by dissociation from plates with 0.25% trypsin–EDTA and passages 2–5 were used in the experiments. The purity of PASMCs was examined by immunofluorescence staining for α‐smooth muscle actin (>95% of cells stained positive) and lack of staining for vimentin.^1^

### 2.3 MTT assay

Cells were seeded at a density of 3 × 10^3^ cells per well in 96 well tissue culture plates. After serum starvation for 24 h, cells were treated with different concentrations of BTZ to detect the toxicity of BTZ to PASMCs. In addition, effects of BTZ in FBS‐, Ang II‐, and PDGF‐BB‐stimulated proliferation were also detected. Cells were pre‐treated with or without BTZ for 1 h, followed by treatment with FBS (10%), Ang II (100 nM), and PDGF‐BB (20 ng/mL) for 24 h. MTT solution was added to the medium for 2 h. The culture medium was then removed and the cells were dissolved in isopropanol and shaken for 10 min. The amount of MTT formazan was quantified at absorbance of 540 and 630 nm using an ELISA reader (DYNEX Technologies, Denkendorf, Germany).

### 2.4 Boyden chamber assay

Migration was assessed with a 24‐well Boyden chamber system (Cat #353097, Corning Costar, Cambridge, MA, USA) with 8 μm pores. Cells were seeded at a density of 5 × 10^4^ cells per well in the upper chamber. After serum starvation for 24 h, FBS (10%), Ang II (100 nM), and PDGF‐BB (20 ng/mL) were added to medium for 24 h, with or without pre‐treatment of BTZ for 1 h in the lower chamber. Non‐migrated cells on the upper membrane surface were removed and those on the lower surface were fixed in methanol and stained with Giemsa (Cat #GS500, Sigma‐Aldrich, Inc., St. Louis, MO, USA). The cell numbers per six high‐power fields (200× HPF) were counted and the mean numbers of cells were used to express migration ability.

### 2.5 Wound healing assay

Cells were seeded at a density of 2 × 10^5^ cells per well in 6 well tissue culture plates. Vertical lines were scratched using a 200 μL pipette tip, and the floating cells were removed by washing three times in 1× PBS. After serum starvation for 24 h, cells were pre‐treated with or without BTZ for 1 h, followed by treatment with FBS (10%), Ang II (100 nM), and PDGF‐BB (20 ng/mL) for 24 h and images were acquired by a light microscopy (Nikon Ti2‐U). Quantitative calculations were performed using Image J software.

### 2.6 DCFH‐DA assay

Cells were seeded at a density of 5 × 10^3^ cells per well in 96‐well tissue culture plates. After serum starvation for 24 h, cells were pre‐treated with or without BTZ for 1 h, followed by treatment with FBS (10%), Ang II (100 nM), and PDGF‐BB (20 ng/mL) for 24 h. Cells were stained with 5 μM of DCFH‐DA for 30 min at 37°C, and then immediately examined under a fluorescence microscope (Nikon Ti2‐U) at an excitation/emission of 485/535 nm. Results were presented as relative fluorescence intensity normalized to control.

### 2.7 MitoSOX assay

Cells were seeded at a density of 5 × 10^3^ cells per well in 96‐well tissue culture plates. After serum starvation for 24 h, cells were pre‐treated with or without BTZ for 1 h, followed by treatment with FBS (10%), Ang II (100 nM), and PDGF‐BB (20 ng/mL) for 24 h. Cells were stained with 5 μM of MitoSOX, a fluorescent probe specific for mitochondrial superoxide, for 1 h at 37°C, and fixed by 1% formaldehyde after washed with PBS. Fluorescence was examined under a fluorescent microscope (Nikon Ti2‐U) at an excitation/emission of 510/580 nm. Results were presented as relative fluorescence intensity normalized to control.

### 2.8 Western blot

Cells were washed by cold 1× PBS, and then lysed by lysis buffer (Cat #78501, M‐PER™, Mammalian Protein Extraction Reagent, Pierce, USA). The lysed cells were centrifuged at 13,000× g for 30 min at 4°C, then, supernatant proteins were collected and stored at −80°C until analysis. Equivalent amount of protein (25 μg/mL) was loaded on SDS–polyacrylamide gel electrophoresis (10%–14%) and transferred to polyvinylidene difluoride membranes. Membranes were incubated in a blocking buffer (5% nonfat dry milk) for 1 h at room temperature, followed by incubation in primary antibody for 2 h at room temperature. After washing off the primary antibody, the blots were incubated with horseradish peroxidase‐linked secondary antibody (Chemicon, Inc., Temecula, CA) for 1 h at room temperature, and the immuno‐reactive bands were detected by chemiluminescence reagents (Cat #NEL103E001EA, PerkinElmer Life Sciences, Inc., Waltham, MA). β‐actin was probed as a control to ensure equal protein loading. All primary antibodies were used at a dilution of 1:1000, and secondary antibodies were used at a dilution of 1:5000 in Western blot.

### 2.9 Statistical analysis

Data were analyzed by GraphPad Prism5.0 (GraphPad Software, Inc., San Diego, CA, USA). One‐way ANOVA was used to compare the means of different groups and the Tukey multiple comparison test was used as a post hoc test following ANOVA. Data are shown as means ± standard error (SEM) from at least three independent experiments. *p* < 0.05 was considered statistically significant.

## 3 RESULTS

### 3.1 BTZ attenuated FBS‐, Ang II‐, and PDGF‐BB‐induced proliferation of PASMCs

**FIGURE 1:**
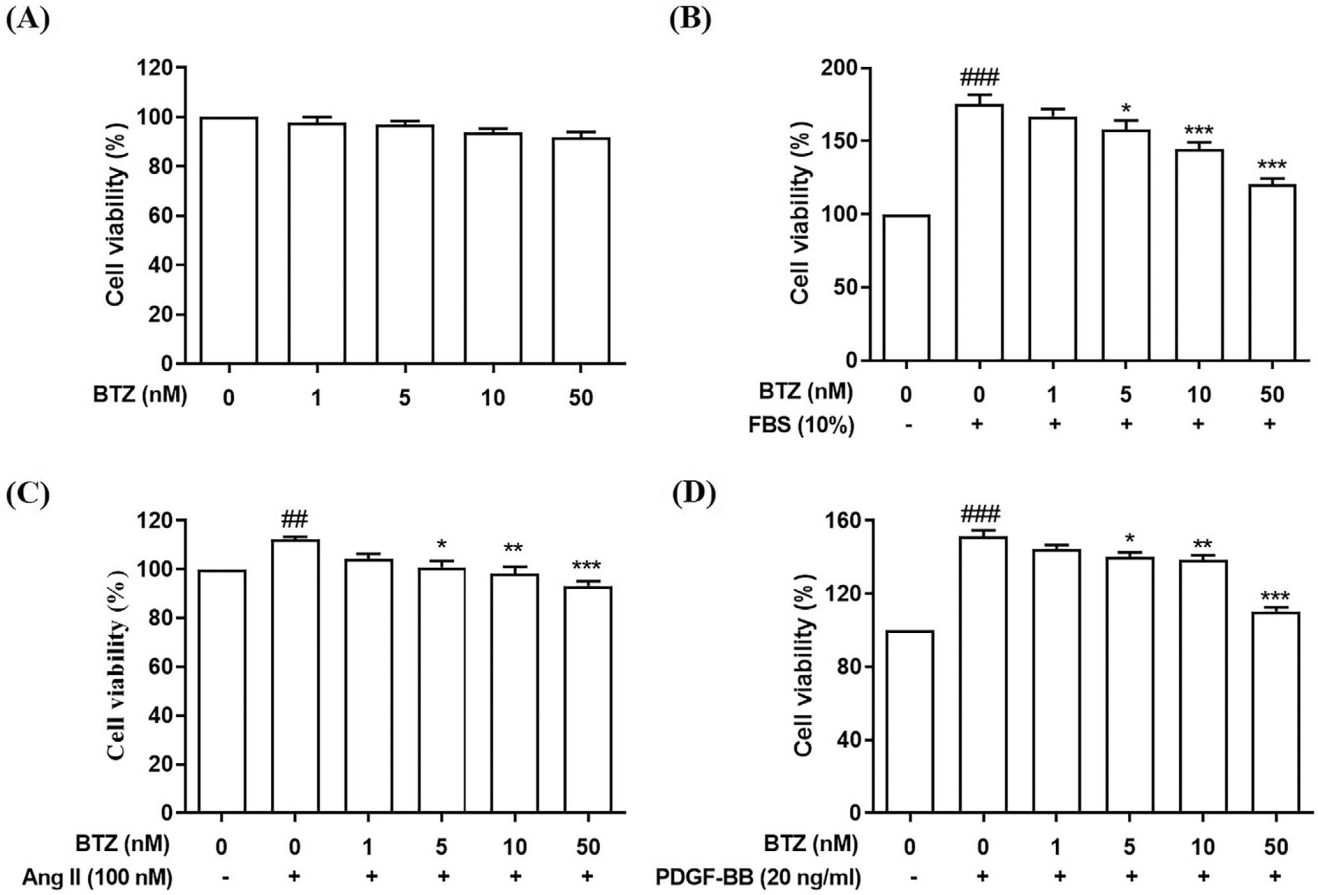
BTZ attenuated FBS‐, Ang II‐, and PDGF‐BB‐induced proliferation of PASMCs. (A) PASMCs were treated with BTZ for 24 h, and then cell viability was detected by MTT assay. Values represent mean ± SEM, *n* = 5. PASMCs were pretreated with BTZ (0–50 nM) for 1 h, followed by treatment with (B) FBS (10%), (C) Ang II (100 nM) and (D) PDGF‐BB (20 ng/mL) for 24 h, and then cell viability was detected by MTT assay. Values represent mean ± SEM, *n* = 5. ##*p* < 0.01, ###*p* < 0.001, compared with the control group. *p < 0.05, **p < 0.01, ***p < 0.001 compared with the induced groups (FBS, Ang II, and PDGF‐BB).

Ang II and PDGF‐BB has been implicated as a key mediator in pulmonary vascular remodeling, contributing essentially to the progression of PAH.^20^, ^21^, ^22^ To investigate effects of BTZ on Ang II and PDGF‐BB‐induced proliferation, cell viability was detected by MTT assay. PASMCs were treated with different concentrations of BTZ for 24 h. MTT assay showed that 0–50 nM of BTZ has no obvious toxicity to PASMCs (Figure 1A). PASMCs were pre‐treated with 0–50 nM of BTZ for 1 h, followed by treatment with FBS (10%), Ang II (100 nM) and PDGF‐BB (20 ng/mL) for 24 h. Results showed that BTZ significantly inhibited FBS‐, Ang II‐ or PDGF‐BB‐induced proliferation (Figure 1B–D).

### 3.2 BTZ attenuated FBS‐, Ang II‐, and PDGF‐BB‐induced migration of PASMCs

**FIGURE 2:**
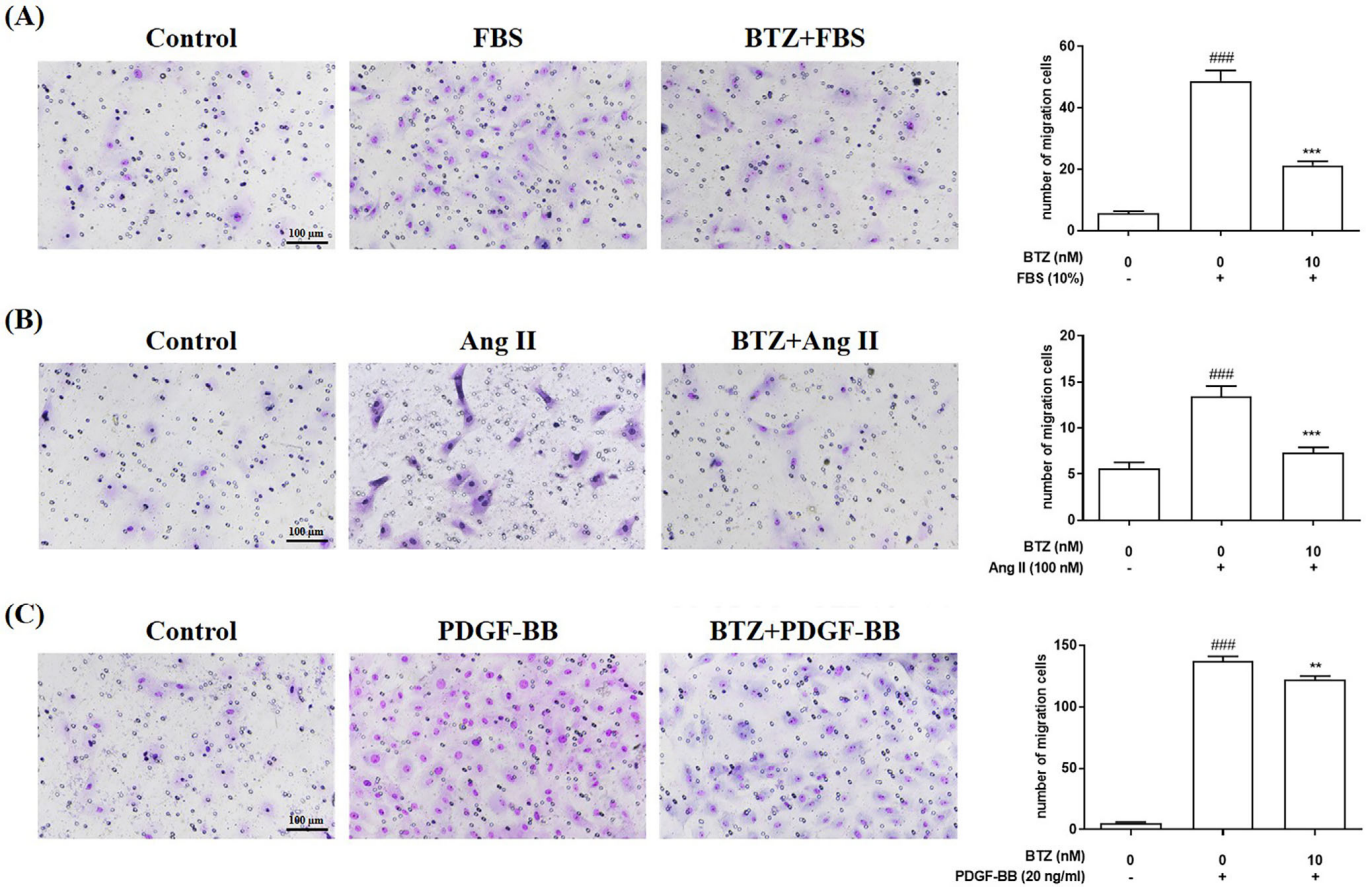
BTZ attenuated FBS‐, Ang II‐, and PDGF‐BB‐induced migration of PASMCs. PASMCs were pretreated with or without BTZ (10 nM) for 1 h, followed by treatment with (A) FBS (10%), (B) Ang II (100 nM), and (C) PDGF‐BB (20 ng/mL) for 24 h, and then migration ability was detected by Boyden chamber assay. Values represent mean ± SEM, *n* = 3. ###*p* < 0.001, compared with the control group. **p < 0.01, ***p < 0.001, compared with the induced groups (FBS, Ang II, and PDGF‐BB).

**FIGURE 3:**
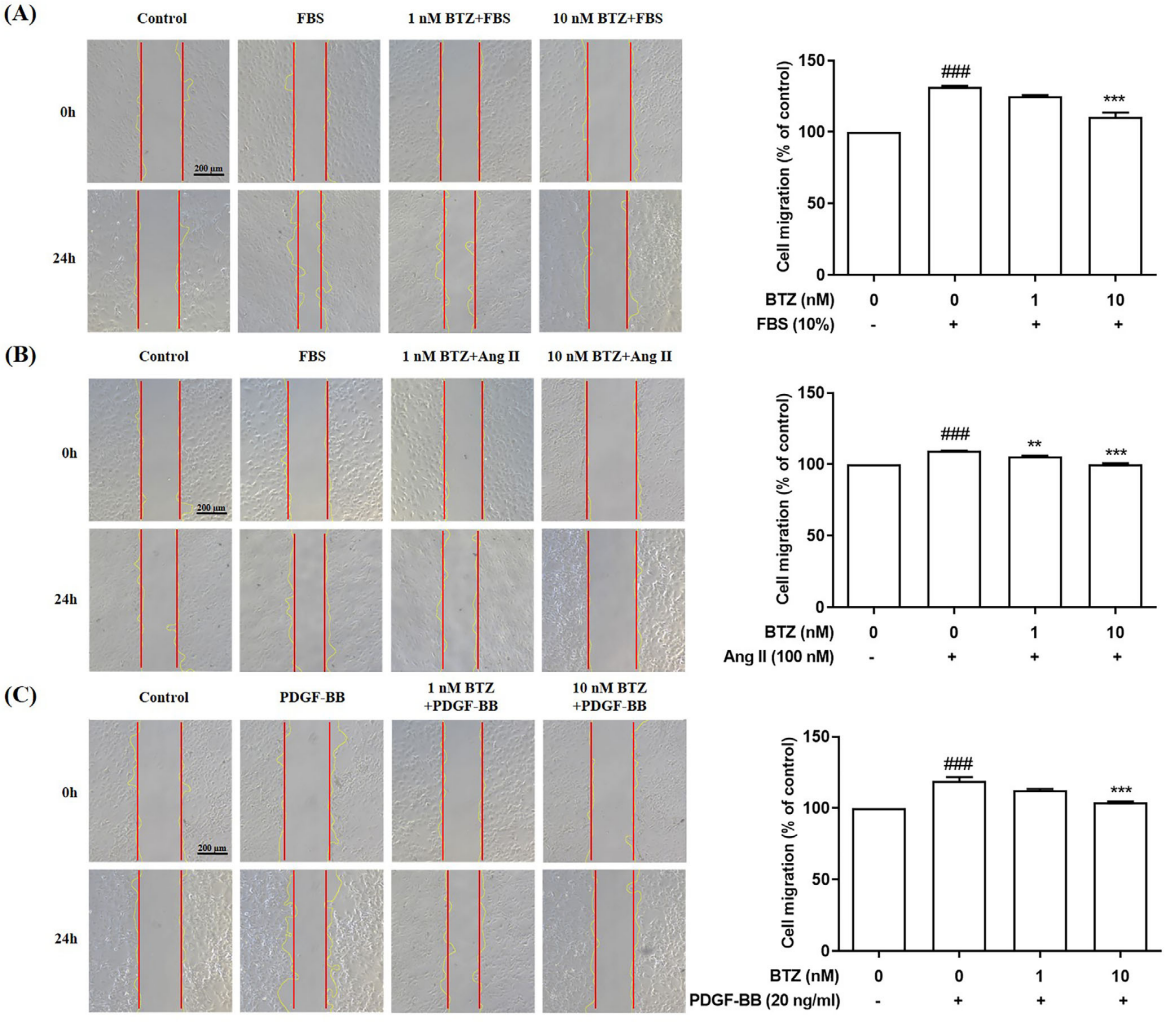
BTZ attenuated FBS‐, Ang II‐, and PDGF‐BB‐induced wound healing migration of PASMCs. PASMCs were pretreated with or without BTZ (10 nM) for 1 h, followed by treatment with (A) FBS (10%), (B) Ang II (100 nM), and (C) PDGF‐BB (20 ng/mL) for 24 h, and then migration ability was detected by wound healing assay. Values represent mean ± SEM, *n* = 3. ###*p* < 0.001, compared with the control group. **p < 0.01, ***p < 0.001, compared with the induced groups (FBS, Ang II, and PDGF‐BB).

Ang II and PDGF‐BB are also known to induce migration of PASMC, contributing to pulmonary vascular remodeling.^20^, ^21^, ^22^ To investigate effects of BTZ on Ang II and PDGF‐BB‐induced migration, Boyden Chamber assay and wound healing assay were used to detect cell migration ability. PASMCs were pre‐treated with BTZ (10 nM) for 1 h, followed by treatment with FBS (10%), Ang II (100 nM) and PDGF‐BB (20 ng/mL) for 24 h, and migration ability was examined. Results showed that the number of migrated cells in Boyden Chamber assay was significantly higher in induced groups (FBS, Ang II, and PDGF‐BB) than that in the control group, and BTZ reduced FBS‐, Ang II‐, and PDGF‐BB‐induced numbers of migrated cells (Figure 2). In wound healing assay, the wound closure rate was also significantly higher in induced groups (FBS, Ang II, and PDGF‐BB) than that in the control group, and BTZ decreased FBS‐, Ang II‐, and PDGF‐BB‐induced wound closure rate (Figure 3). These results indicated that BTZ significantly attenuated FBS‐, Ang II‐, and PDGF‐BB‐induced migration of PASMCs.

### 3.3 BTZ attenuated FBS‐, Ang II‐, and PDGF‐BB‐induced ROS production of PASMCs

**FIGURE 4:**
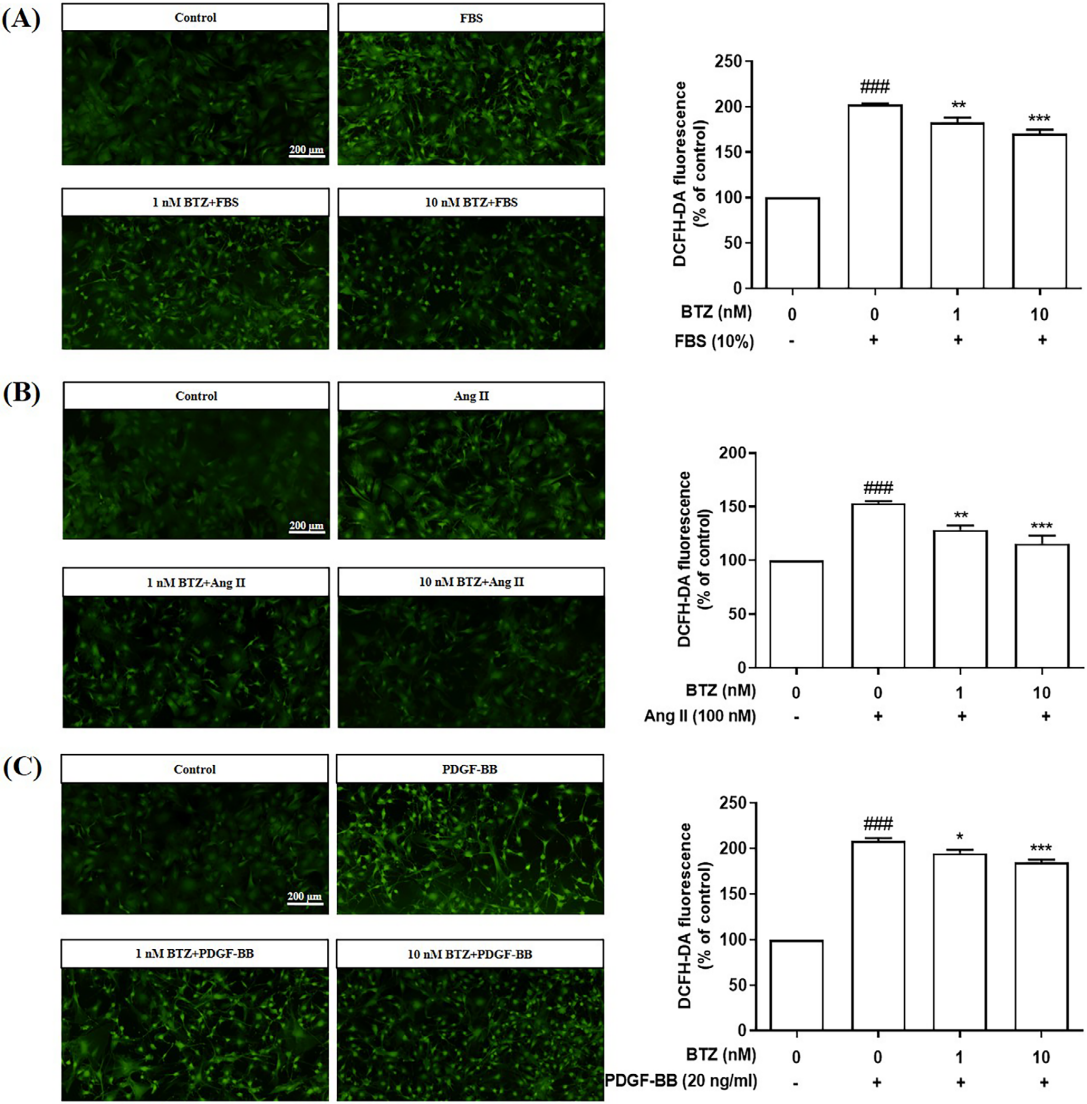
BTZ attenuated FBS‐, Ang II‐, and PDGF‐BB‐induced ROS production in PASMCs. PASMCs were pretreated with BTZ for 1 h, followed by treatment with (A) FBS (10%), (B) Ang II (100 nM), and (C) PDGF‐BB (20 ng/mL) for 24 h, and then ROS production was detected by DCFH‐DA assay. Values represent mean ± SEM, *n* = 6. ###*p* < 0.001, compared with the control group. *p < 0.05, **p < 0.01, ***p < 0.001, compared with the induced groups (FBS, Ang II, and PDGF‐BB).

**FIGURE 5:**
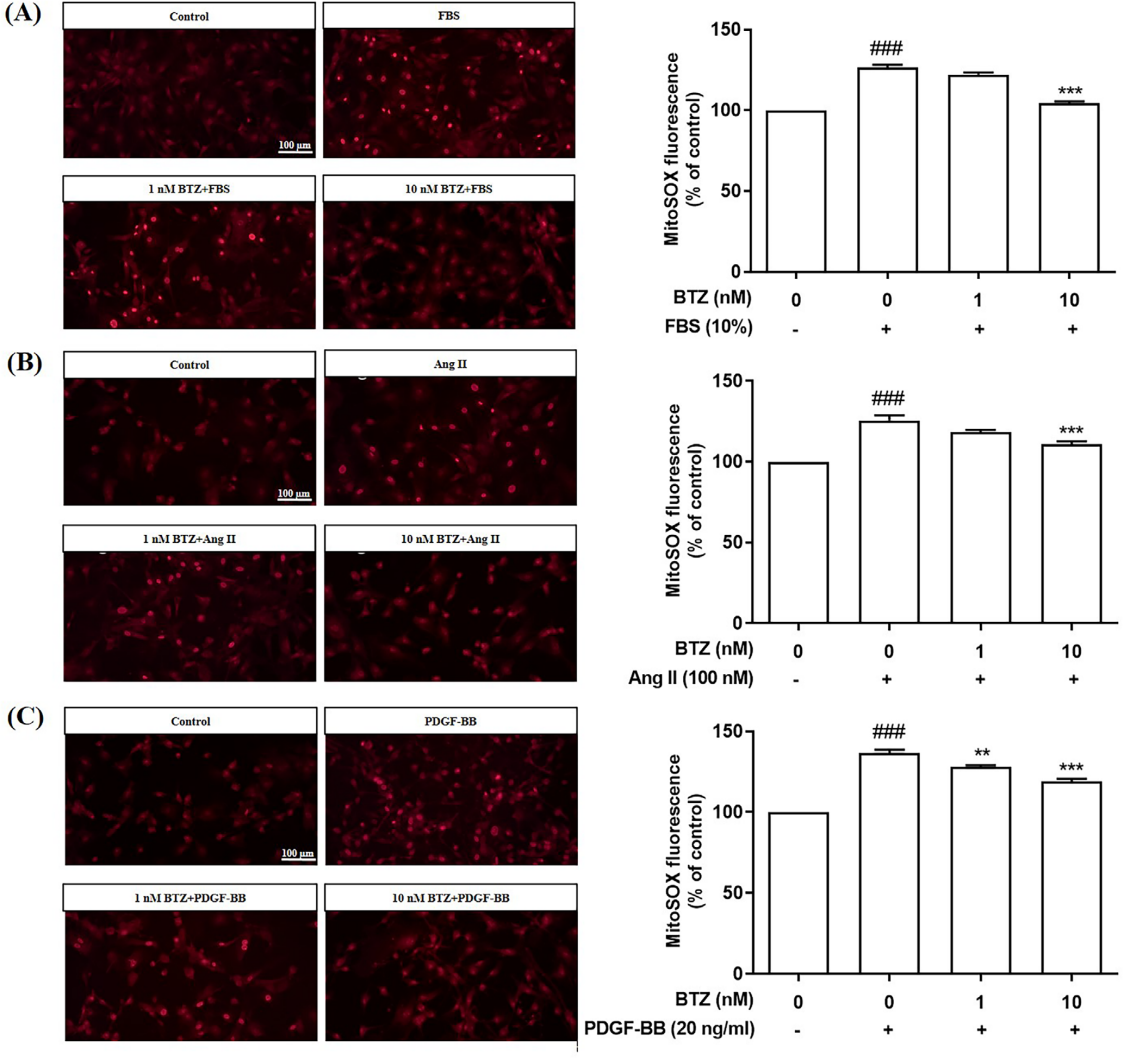
BTZ attenuated FBS‐, Ang II‐, and PDGF‐BB‐induced mitochondrial ROS production in PASMCs. PASMCs were pretreated with BTZ for 1 h, followed by treatment with (A) FBS (10%), (B) Ang II (100 nM), and (C) PDGF‐BB (20 ng/mL) for 24 h, and then mitochondrial ROS production was detected by MitoSOX assay. Values represent mean ± SEM, *n* = 6. ###*p* < 0.001, compared with the control group. **p < 0.01, ***p < 0.001, compared with the induced groups (FBS, Ang II, and PDGF‐BB).

Excessive ROS production causes oxidative stress, contributing to the pathogenesis of PAH.^6^ To investigate effects of BTZ on ROS production, cellular ROS production and mitochondrial ROS production were detected by DCFH‐DA assay and MitoSOX assay, respectively. Furthermore, PASMCs were pre‐treated with BTZ (10 nM) for 1 h, followed by treatment with FBS (10%), Ang II (100 nM) and PDGF‐BB (20 ng/mL) for 24 h, and then ROS production was detected. Results showed that BTZ attenuated FBS‐, Ang II‐, and PDGF‐BB‐induced cellular ROS production (Figure 4). Similarly, BTZ also attenuated FBS‐, Ang II‐, and PDGF‐BB‐induced mitochondrial ROS production (Figure 5). Furthermore, we found that NAC, a ROS scavenger, attenuated FBS‐, Ang II‐, and PDGF‐BB‐induced cellular ROS production and mitochondrial ROS production. These results indicate that BTZ has similar ROS‐reducing effects as NAC.

### 3.4 BTZ attenuated Ang II‐ and PDGF‐BB‐induced the phosphorylation of ERK and Akt in PASMCs

**FIGURE 6:**
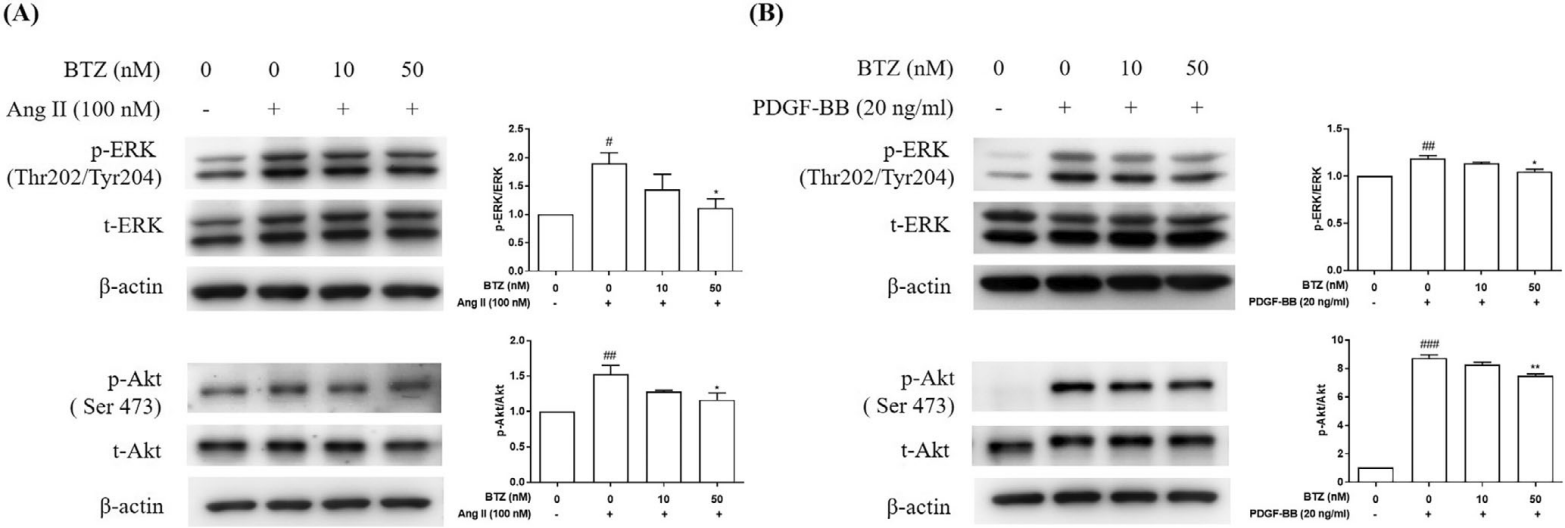
BTZ attenuated Ang II‐ and PDGF‐BB‐induced phosphorylation of ERK and Akt in PASMCs. PASMCs were pretreated with BTZ for 1 h, followed by treatment with (A) Ang II (100 nM) and (B) PDGF‐BB (20 ng/mL) for 15 min, and then the phosphorylated and total forms of ERK and Akt was detected by Western Blot. Value represents the mean ± SEM, *n* = 3. #*p* < 0.05, ##*p* < 0.01, ###*p* < 0.001, compared with the control group. *p < 0.05, **p < 0.01 compared with the induced groups (Ang II and PDGF‐BB).

**FIGURE 7:**
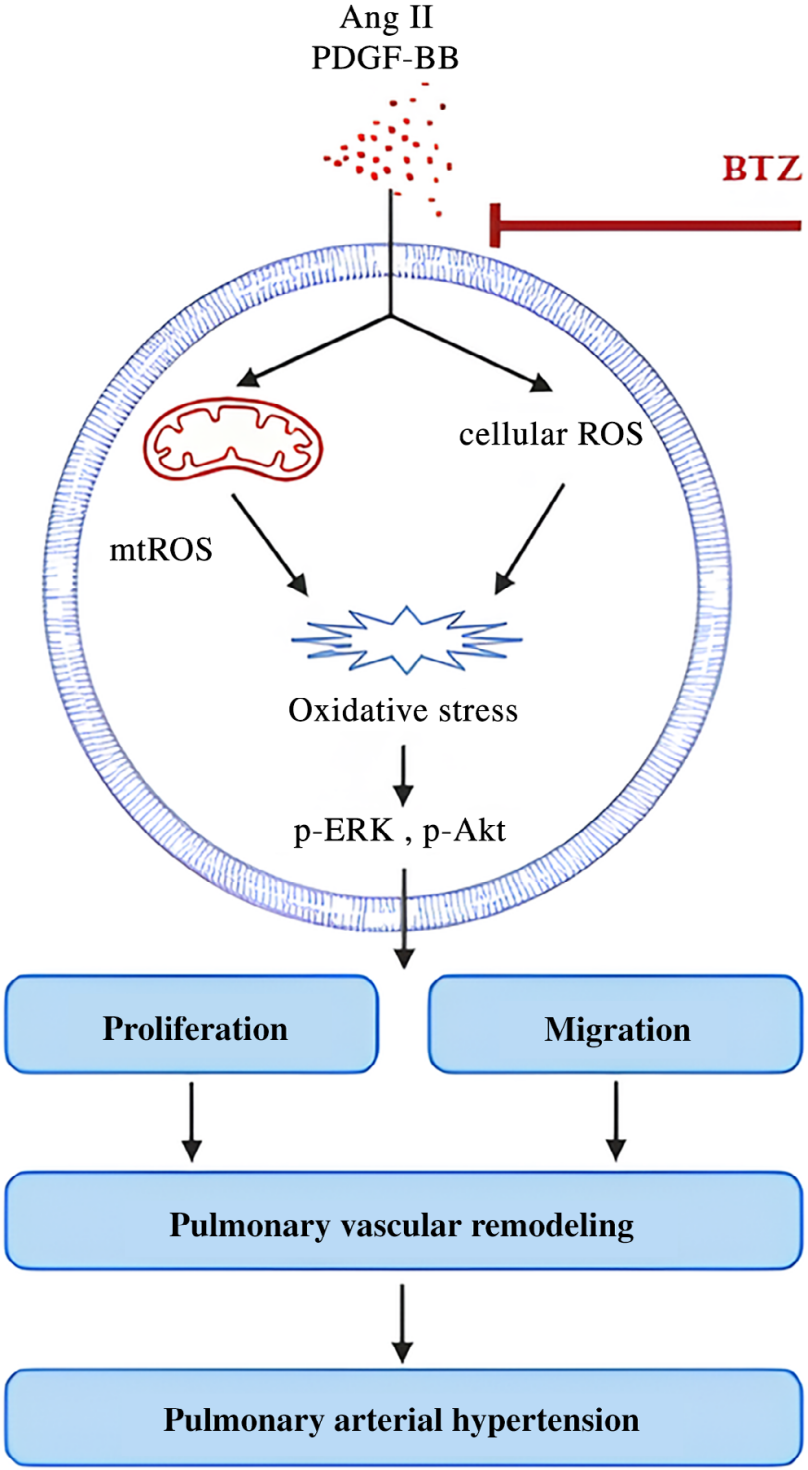
Proposed diagram of BTZ attenuating Ang II‐ and PDGF‐BB‐induced proliferation and migration of PASMCs. BTZ attenuates proliferation, migration, ROS production, and the phosphorylation of ERK and Akt induced by Ang II and PDGF‐BB in PASMCs, suggesting a possible therapeutic role of BTZ in pulmonary vascular remodeling and providing novel therapeutic strategies for PAH. mtROS, mitochondrial ROS.

ERK and Akt activation play important roles in vascular remodeling.^23^ To investigate effects of BTZ on ERK and Akt activation, the phosphorylation of ERK and Akt was detected by Western Blot. PASMCs were pre‐treated with BTZ (10 or 50 nM) for 1 h, followed by treatment with Ang II (100 nM) and PDGF‐BB (20 ng/mL) for 15 min, and the phosphorylation of ERK and Akt was detected. Results showed that BTZ inhibited Ang II‐ and PDGF‐BB‐induced phosphorylation of ERK and Akt in PASMCs (Figure 6). In summary, a proposed mechanism in this study is shown in Figure 7.

## 4 DISCUSSION

Abnormal proliferation and migration of vascular smooth muscle cells are characteristic of many proliferative vascular diseases such as PAH, atherosclerosis, and restenosis. In addition, targeting oxidative stress is considered as a preventive and therapeutic approach for vascular remodeling.^24^ Previous studies have demonstrated that Ang II and PDGF‐BB induce the proliferation, migration, and ROS production in PASMCs.^20^, ^21^, ^22^ In this study we demonstrated that BTZ attenuated FBS‐, Ang II‐ and PDGF‐BB‐induced proliferation, migration, and ROS production. In addition, the phosphorylation of ERK and Akt induced by Ang II and PDGF‐BB was also inhibited by BTZ in PASMCs. Our study revealed that BTZ attenuated Ang II and PDGF‐BB‐induced proliferation, migration, and ROS production, which was associated with down‐regulation of ERK and Akt in PASMCs.

The imbalance between ROS production and the antioxidant capacity of cells results in oxidative stress, contributing to the pathogenesis of PAH.^6^ ROS production appears to be critical for proliferation and migration of smooth muscle cells and is essential in vascular remodeling of PAH.^1^, ^25^ There are many sources of ROS, including the mitochondrial respiratory chain, the NADPH oxidases, xanthine oxidase, lipoxygenases, and nitric oxide synthases.^25^ Mitochondria and NADPH oxidases are the major sources of cellular ROS production in vascular smooth muscle cells,^25^, ^26^ and approximately 90% of cellular ROS production is generated in mitochondria^26^, ^27^; therefore, we focused on effects of BTZ on mitochondrial ROS production in this study.

There was contradictory effect of anti‐oxidative in different cells or organs. Some previous studies have shown that in cancer cell lines BTZ can induce ROS^28^, ^29^; however, in a recent animal study of myocardial ischemia reperfusion injury, BTZ could reduce ROS by augmenting oxidative stress related protein levels of superoxide dismutase, catalase and glutathione. Mechanistically, they found that BTZ promoted nuclear translocation of transcriptional factor nuclear factor erythroid 2‐related factor 2 and heme oxygenase‐1 expression.^30^ In our study, BTZ attenuated ROS production induced by three inducers in PASMCs, suggesting cell‐specific differences in BTZ's effects on ROS.

BTZ is an anti‐cancer agent that has been reported to induce endoplasmic reticulum (ER) stress, to activate unfolded protein response, and trigger apoptosis and autophagy in several cancer cells. Currently, there is no study investigates that BTZ treatment can induce ER stress or unfolded protein responses in the PASMCs, however, ROS mediates several critical aspects of the ER stress response. Of the many cell types, myeloma cells rely heavily on the secretory pathway of ER, which is sensitive to proteasomal inhibition, so appear to be particularly sensitive to BTZ. Recent reports indicate that proteasome inhibitors have diverse effects on nonmalignant cells, and their effects depend on dosage and cell type. Biphasic dosage effects have been reported for proteasome inhibitors in different cells, including astrocytes and endothelial cells, whereby they afford protection at low doses and induce apoptosis at high doses.^31^

ERK and Akt activation play important roles in cell proliferation, and has been increasingly recognized as a regulator of vascular remodeling.^23^ Previous studies have shown that the expression levels of phosphorylated ERK and Akt was upregulated with Ang II and PDGF‐BB treatment, which resulted in the promotion of PASMCs proliferation.^1^, ^32^ Our results showed that BTZ attenuated Ang II‐ and PDGF‐BB‐induced phosphorylation of ERK and Akt, suggesting that BTZ inhibited proliferation and migration, at least in part through the ERK and Akt pathways.

Several studies have also investigated the possible mechanisms by which BTZ regulates cell proliferation and migration. A previous study indicates that BTZ attenuates Ang II‐induced hypertensive response in rats, with associated decreased aortic wall‐to‐lumen ratio, collagen deposition, MMP2 activity, proteasomal chymotrypsin‐like activity, Ki67 staining, ROS generation, VCAM‐1 immunoreactivity, and expression of TIMP1 and TIMP2.^33^ In PASMCs, our previous study suggests that BTZ inhibits proliferation by overexpression of mitofusin‐2.^2^ Likewise, BTZ inhibits proliferation by inhibiting caveolin‐1/calcium signaling axis in human PASMCs.^34^ In addition, BTZ reduces proliferation and migration of retinal pigment epithelium cells by the NF‐κB signaling pathway.^35^ Compared with previous studies in various cells, our study shows that in PASMCs, BTZ also conveys anti‐proliferative and anti‐migratory effects. Furthermore, the present study shows novel mechanisms underlying these anti‐remodeling effects by BTZ, including attenuation of mitochondrial ROS production and deactivation of MAPK and Akt.

In addition, despite the apparent success of BTZ in the treatment of multiple myeloma, some patients still do not respond to BTZ therapy or develop drug resistance.^36^ BTZ inhibits the CT‐L activity, but does not have any significant effect on T‐L and CP‐L activity.^37^ Inhibition of CT‐L by BTZ leads to the compensatory activation of T‐L and CP‐L, resulting in resistance to BTZ. The development of an irreversible pan‐proteasome inhibitor is considered an effective approach to overcome drug resistance.^2^ For example, carfilzomib is a next‐generation FDA‐approved proteasome inhibitor and exerts its inhibitory function by binding to the CT‐L subunit.^38^ Unlike BTZ, the binding of carfilzomib to the CT‐L subunit is irreversible, and carfilzomib maintains its cytotoxic potential in the BTZ‐resistant cell lines.^39^ In addition, marizomib is another next‐generation irreversible proteasome inhibitor.^37^ As marizomib inhibits all three major proteolytic activities (CT‐L activity, T‐L activity and CP‐L activity), it can target proteasomes more broadly.^6^, ^40^ Marizomib has been tested in clinical trials in a variety of cancers such as refractory multiple myeloma, leukemia, lymphoma, glioblastoma, and malignant glioma.^6^ Whether the newly developed next‐generation proteasome inhibitors have more potential than BTZ in the treatment of PAH deserves further study in the future.

There are two limitations in this study. First, there is no animal experiment in this study. However, previous studies have shown that in animal models of mice and rats, BTZ could attenuate right ventricular systolic pressure, suppress right ventricular hypertrophy and thickening of pulmonary vascular walls.^16^, ^19^ Thus in our in vitro study, we aimed to investigating cellular and molecular mechanisms underlying its anti‐remodeling effects. Second, we did not compare the efficacy of BTZ with NAC in the same experiments, since our study design was not to determine if BTZ is the best ROS‐reducing agent. Instead, we assume that in addition to ROS‐reduction, there are other potential synergistic mechanisms, such as signal transduction, underlying the anti‐remodeling effects of BTZ and may warrant further investigation.

In conclusion, our study demonstrated the therapeutic potential and mechanism of proteasome inhibition in Ang II‐ and PDGF‐BB‐induced dysregulation of PASMCs, shedding some lights in novel therapeutic strategies for PAH. Further clinical investigations are required to substantiate these important findings.

## CONFLICT OF INTEREST STATEMENT

The authors declare no conflicts of interest.

## ETHICS STATEMENT

All procedures involved in this study were approved by the Animal Care and Use Committee of the Kaohsiung Medical University (IACUC Approval No: 108118).
